# Methylation of *BRCA1* Promoter Region Is Associated with Unfavorable Prognosis in Women with Early-Stage Breast Cancer

**DOI:** 10.1371/journal.pone.0056256

**Published:** 2013-02-06

**Authors:** Nicholas C. Hsu, Ya-Fang Huang, Kazunari K. Yokoyama, Pei-Yi Chu, Fang-Ming Chen, Ming-Feng Hou

**Affiliations:** 1 Graduate Institute of Medicine, Kaohsiung Medical University, Kaohsiung, Taiwan; 2 Cancer Center, Kaohsiung Medical University Hospital, Kaohsiung, Taiwan; 3 Department of Pathology, St. Martin De Porres Hospital, Chiayi, Taiwan; 4 Division of General and Gastroenterological Surgery, Department of Surgery, Kaohsiung Medical University Hospital, Kaohsiung, Taiwan; 5 National Sun Yat-Sen University-Kaohsiung Medical University Joint Research Center, Kaohsiung, Taiwan; 6 Kaohsiung Municipal Ta-Tung Hospital, Kaohsiung, Taiwan; King Faisal Specialist Hospital & Research Center, Saudi Arabia

## Abstract

*BRCA1*-associated breast cancers are associated with particular features such as early onset, poor histological differentiation, and hormone receptor negativity. Previous studies conducted in Taiwanese population showed that the mutation of *BRCA1* gene does not play a significant role in the occurrence of breast cancer. The present study explored methylation of *BRCA1* promoter and its relationship to clinical features and outcome in Taiwanese breast cancer patients. Tumor specimens from a cohort of 139 early-stage breast cancer patients were obtained during surgery before adjuvant treatment for DNA extraction. Methylation of *BRCA1* promoter region was determined by methylation-specific PCR and the results were related to clinical features and outcome of patients using statistical analysis. Methylation of the *BRCA1* promoter was detected in 78 (56%) of the 139 tumors. Chi-square analysis indicated that *BRCA1* promoter methylation correlated significantly with triple-negative (ER-/PR-/HER2-) status of breast cancer patients (*p* = 0.041). The Kaplan-Meier method showed that *BRCA1* promoter methylation was significantly associated with poor overall survival (*p* = 0.026) and disease-free survival (*p* = 0.001). Multivariate analysis which incorporated variables of patients' age, tumor size, grade, and lymph node metastasis revealed that BRCA1 promoter methylation was associated with overall survival (*p* = 0.27; hazard ratio, 16.38) and disease-free survival (*p* = 0.003; hazard ratio, 12.19). Our findings underscore the clinical relevance of the methylation of *BRCA1* promoter in Taiwanese patients with early-stage breast cancer.

## Introduction

Breast cancer is the most common type of cancer and ranked fourth in cancer mortality in Taiwan [1]. Incidence of breast cancer in Taiwan has been increasing steadily and the rise can be largely attributed to the rise of newly diagnosed young patients [2]. It has been speculated that the early onset of breast cancer in Taiwanese women may be explained by the combination of Westernized diet, sedentary lifestyle, delay childbearing, and exposure to environmental chemicals.

*BRCA1* is a major cancer predisposition gene that has been the subject of intense investigation since it was identified and cloned in 1994 [3]. Breast tumors arising from *BRCA1* mutation acquired distinct pathological and gene expression profiles [4]. The spectrum of *BRCA1* gene mutations in breast cancer patients in various populations has been investigated [5]–[10]. Studies conducted in Taiwan had suggested that the mutation of *BRCA1* contributes little to the occurrence of breast cancer [11], [12].

Despite germ-line mutations in *BRCA1* can account for a significant fraction of familial breast cancer cases, a large proportion of familial breast cancer are not related to mutations in *BRCA1* or *BRCA2* [13]–[16]. Hypermethylation of cytosine residues in CpG islands within the promoter of many tumor suppressor genes is strongly correlated with the absence of gene expression. The epigenetic transcriptional silencing provides an alternative mechanism for the loss of function of tumor suppressor gene during cancer development [17], [18]. Hypermethylation of the *BRCA1* promoter has been reported in breast cancer with links to down-regulated mRNA and protein level in tumors and cell lines [19]–[23]. Aberrant *BRCA1* promoter methylation has also been found to be associated with particular biological and clinicopathological features [24]–[27]. However these reports did not result in similar conclusion as the limited population examined and the high heterogeneity among breast cancer patients in these studies might lead to the inconclusive finding. The aim of the current study was to examine the prevalence and clinical relevance of *BRCA1* promoter methylation in Taiwanese women with breast cancer. Our results indicated that promoter region of *BRCA1* gene is frequently methylated in Taiwanese patients with early-stage breast cancer. *BRCA1*-methylated tumor exhibited poorer survival outcome.

## Patients and Methods

### Ethics Statement

The study was approved by the institutional review board of Kaohsiung Medical University Hospital, Taiwan. Written informed consent was obtained from all participants.

### Study subjects

Surgically resected specimens from a cohort of one hundred thirty-nine patients with early-stage breast cancer (stages I and IIA) were collected at Kaohsiung Medical University Hospital between October 2007 and July 2009. Tissues were frozen at −80°C until the extraction of DNA. Genomic DNA was extracted using the QIAamp DNA Mini Kit (Qiagen, Valencia, CA, USA) according to the manufacturer's instructions.

### Methylation-specific PCR

One µg of genomic DNA was modified using a CpGenomet DNA modification kit (Chemicon, Temecula, CA) according to the manufacturer's protocol and resuspended in TE buffer. Modified DNA was amplified in a total volume of 10 µL solution containing 1x PCR buffer, 1.5 mM MgCl_2_, 200 ng of each primer, 0.2 mM of each dNTP and 1 U Platinum Taq Polymerase (Life Technologies, Carlsbad, CA). Primers and conditions used for the methylation-specific PCR were first developed by Esteller *et al* [19]. Primer sequences for unmethylated PCR were 5′- TTG GTT TTT GTG GTA ATG GAA AAG TGT-3' and 5'- CAA AAA ATC TCA ACA AAC TCA CAC CA-3' and primers for methylated reaction were 5'- TCG TGG TAA CGG AAA AGC GC-3' and 5'- AAA TCT CAA CGA ACT CAC GCC G-3'. The sense primers of the unmethylated PCR and methylated PCR begin at nucleotide position 1536 and 1543 bp of *BRCA1* (GenBank sequence U37574), respectively. This region crosses the major transcription start site of *BRCA1* gene [28].The amplified product of unmethylated PCR is 86 bp and that of methylated product is 75 bp.

For methylated genes, SssI treated DNA (MDA-MB-231 cells) was used as a positive control and DNA from normal lymphocytes was used as a negative control. After amplification, PCR products were then loaded and electrophoresed on 2% agarose gels, stained with ethidium bromide and visualized under UV illumination. The presence of a product in the methylated reaction indicated the presence of methylated *BRCA1* genes. Tumors that were positive for both methylated and unmethylated reactions were classified as having methylated *BRCA1* genes.

### Immunohistochemical staining

Immunohistochemical staining was performed on representative samples. The selected paraffin-embedded sections were cut and mounted on poly-*l*-lysine-coated slides. Staining was carried out with a mouse monoclonal BRCA1 antibody (Clone MS110, Biocare Medical, Concord, CA) at 1∶50 dilution in a Leica Bond-max automated immunostainer (Leica Microsystems, Newcastle, UK), according to manufacturer's protocol.

### Statistical analysis

Association between *BRCA1* methylation status and clinicopathological characteristics was analyzed by use of Pearson chi-square test. Survival rate and disease recurrence were calculated using Kaplan-Meier analysis and compared by the Cochran-Mantel-Haenszel test. Overall survival (OS) was calculated from the time of initial diagnosis to death of any cause. Disease-free survival (DFS) was defined as the time between initial diagnosis and diseases recurrence. Patients alive or disease free at the end of the follow-up period were censored. Data from medical charts were retrieved, and the patients' outcomes were followed until January, 2012 or date of death, whichever occurred first. Potential confounding factors were adjusted and analyzed by use of the Cox proportional hazards regression model. The variables in the model included age, tumor size, lymph node metastasis, and histological grade. All statistical calculations were done using SPSS version 17.0 for windows (SPSS, Inc., Chicago, IL). A *p* <0.05 was considered significant.

## Results

**Figure 1:**
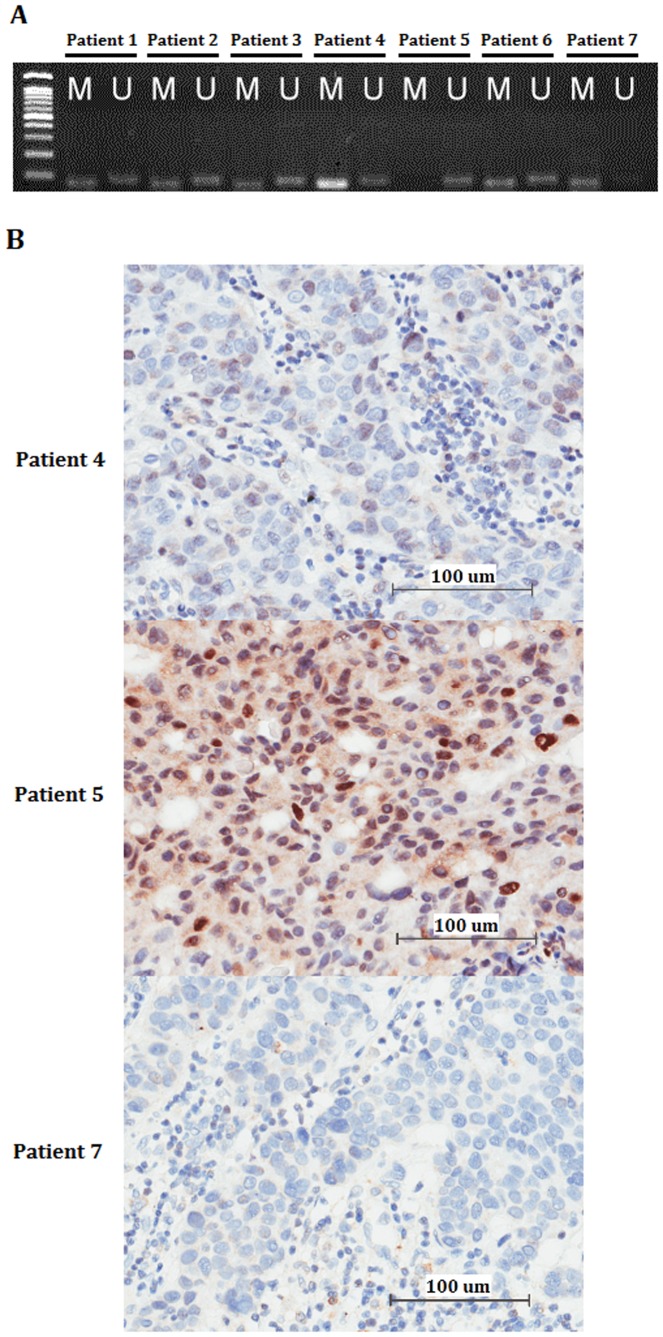
*BRCA1* promoter methylation and immunohistochemical analysis of BRCA1 in early-stage breast tumors. (A) DNA methylation status of the *BRCA1* promoter determined by methylation-specific PCR. M-labeled lanes represent PCR products amplified with methylation-specific primers (75 bp). U-labeled lanes indicate the presence of unmethylated genes (82 bp). Patients 1, 2, 3, 4, 6, and 7 show the presence of a PCR product in both reactions, indicating methylation of the *BRCA1* promoter region. Patients 5 shows unmethylated gene. Molecular weight marker used is a 100-bp ladder. (B) Representative corresponding images of immunohistochemical staining of BRCA1. Scale bars, 100 µm.

The clinical characteristics of the 139 early-stage breast cancer patients at the time of surgery are summarized in Table 1. Among these patients, the medium age was 49 years (ranging from 24 to 71 years). Histologically, all cases were invasive ductal carcinomas. Methylation of the *BRCA1* promoter was detected in 78 (56%) of the 139 tumors examined in which 28 (20%) tumors were positive only for the methylated reaction and 50 (36%) were positive for both unmethylated and methylated reactions. Figure 1A shows representative methylation status of *BRCA1* promoter by methylation-specific PCR. To evaluate the effect of DNA methylation on protein expression, immunohistochemical staining of BRCA1 was carried out. Consistent with the methylation status of *BRCA1* promoter, the expression levels of BRCA1 protein were reduced in tumors with methylated genes (Figure 1B).

**Table 1:** Patient clinical characteristics and *BRCA1* promoter methylation in early-stage breast cancer.

Characteristics	Overall (N = 139)	%	Unmethylated		Methylated		*p*	
			N	%	N	%		
Age								
<45 y	30	21.58	14	22.95	16	20.51	0.749	
45–55 y	62	44.60	25	40.98	37	47.44		
>55 y	47	33.81	22	36.07	25	32.05		
Tumor size								
≤20 mm	105	76.09	48	80.00	57	73.08	0.345	
>20 mm	33	23.91	12	20.00	21	26.92		
Lymph node status								
-	116	83.45	47	77.05	69	88.46	0.072	
+	23	16.55	14	22.95	9	11.54		
Stage								
I	83	59.71	34	55.74	49	62.82	0.398	
IIA	56	40.29	27	44.26	29	37.18		
Grade								
I	15	10.95	8	13.11	7	9.21	0.261	
II	81	59.12	39	63.93	42	55.26		
III	41	29.93	14	22.95	27	35.53		
ER								
-	51	36.96	21	34.43	30	38.96	0.584	
+	87	63.04	40	65.57	47	61.04		
PR								
-	65	47.10	29	47.54	36	46.75	0.927	
+	73	52.90	32	52.46	41	53.25		
HER2								
-	91	65.94	36	59.02	55	71.43	0.127	
+	47	34.06	25	40.98	22	28.57		
Triple-negative breast cancer (ER-/PR-/HER2-)								
Yes	21	15.22	5	8.20	16	20.78		0.041
No	117	84.78	56	91.80	61	79.22		

**Figure 2:**
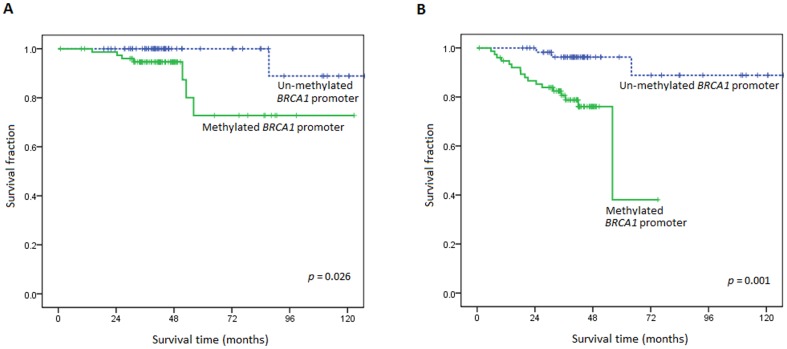
Kaplan-Meier curves of overall survival (A) and disease-free survival (B) for patients with early-stage breast cancer according to *BRCA1* promoter methylation.

Chi-square analysis revealed that despite there was no significant difference between status of *BRCA1* promoter methylation in age, tumor size, lymph node status, stage, histologic grade, ER, PR, and HER-2 positivity. A statistically significant association was found between *BRCA1* promoter methylation and triple-negative breast cancer (ER-/PR-/HER2-) in this cohort of patients (*p* = 0.041). In Cox proportional hazards analysis, *BRCA1* promoter methylation status was analyzed with tumor size, lymph node metastasis, histological grade, and age for their impact on OS and DFS. Adjusted for all these covariates, *BRCA1* promoter methylation was identified as an independent predictor for OS (*p* = 0.027; hazard ratio = 16.38) and DFS (*p* = 0.003; hazard ratio = 12.19) (Table 2). No other clinical variable was found to be independently associated with death in this set of early-stage breast cancer patients. Kaplan-Meier survival curves showed that methylated promoter of the *BRCA1* gene was associated with poor OS (*p* = 0.026; Figure 2A) and DFS (*p* = 0.001; Figure 2B).

**Table 2:** Multivariate analysis of clinical factors and *BRCA1* promoter methylation in patients with early-stage breast cancer for overall survival and disease-free survival.

Variable at baseline	Overall survival					Disease-free survival			
	Hazard Ratio	95.0% CI		*p*		Hazard Ratio		95.0% CI	*p*
*BRCA1* promoter									
Unmethylated									
Methylated	16.38	1.37	195.45	0.027		12.19	2.29	64.75	0.003
Tumor Size									
≤20 mm									
>20 mm	0.88	0.17	4.51	0.878		1.70	0.55	5.21	0.357
Lymph node status									
-									
+	0.23	0.02	3.59	0.297		1.16	0.27	5.10	0.841
Grade									
I/II									
III	3.77	0.68	20.80	0.128		0.95	0.33	2.70	0.917
Age									
≤45 y	0.19	0.02	1.58	0.123		1.19	0.23	6.26	0.841
46–55 y	0.16	0.02	1.20	0.074		0.81	0.17	3.93	0.793
>55 y									

## Discussion

The incidence of breast cancer in Taiwan has increased at a significantly faster pace than western countries over the last two decades [2] and stage 0 to IIA breast cancer currently comprises more than half of all newly diagnosed cases.

*BRCA1* promoter hypermethylation has been implicated as one of the mechanisms of loss of gene expression and has been identified in 9–32% of unselected sporadic breast cancer [19], [25], [28]–[31]. We report here that hypermethylation of the *BRCA1* gene promoter is present in 56% (78 of 139) of Taiwanese women with early-stage sporadic breast carcinomas, which is significantly higher than previously reported frequencies for this alteration in unselected sporadic breast tumors. The incidence of *BRCA1* methylation has previously been reported to be higher in breast tumors of infiltrating ductal type [31]. Since all our patients are of the infiltrating ductal type, this finding was somewhat comparable to that of current literature.

BRCA1 protein expression was found to be absent or markedly decreased in the majority of the *BRCA1* methylated tumors, suggesting epigenetic gene silencing in these tumors [31]. Breast cancers with *BRCA1* promoter methylation also showed decreased expression of ER [25], [29], [31] and basal-like phenotype [25]. Our results indicated that *BRCA1* promoter methylation correlated significantly with triple-negative breast cancer. (*p* = 0.041).

More than half of patients with *BRCA1* mutation have triple-negative breast cancer, and share common clinical and pathological features [10], [32], [33]. However, a significant portion of triple-negative breast cancer patients do not carry *BRCA1* mutations. Alteration in the function of the *BRCA1* gene product has also been identified as an alternative route which also results in impaired BRCA1 function in triple-negative breast cancer [34]. Our finding suggests that *BRCA1* promoter methylation may also have an etiological role on the development of triple-negative phenotype and underlie triple-negative breast cancer.

Given that *BRCA1*-associated breast cancers are more likely to be of high grade [35], or estrogen-receptor negative [35], and p53 positive [36], it has been speculated that *BRCA1*-related breast cancer is more aggressive than sporadic breast cancer. However, most studies conducted in specific populations suggest that survival for women with *BRCA1* mutations is similar to that of women without the mutations [37], [38].

The Kaplan-Meier survival curves of the early-stage breast cancer patients plotted into 2 groups according methylation of *BRCA1* promoter region showed that patients with methylated BRCA1 promoter had a significantly shorter OS and DFS than patients with unmethylated gene. (*p* = 0.026 and *p* = 0.001, respectively). Multivariate analysis adjusted for age, lymph node metastasis, size and grade of tumor also revealed that methylation of the BRCA1 promoter was an independent prognostic marker for survival in early-stage breast cancer (OS, *p* = 0.027; DFS, *p* = 0.003). Our findings suggests that *BRCA1* promoter methylation is a better predictor of recurrence and survival than factors such as tumor size, lymph node metastasis, histological grade, and age in early-stage breast cancer.

In conclusion, this study highlights the frequent promoter methylation of *BRCA1* and its prognostic significance, irrespective of *BRCA1* gene mutation in Taiwanese patients with early-stage breast cancer. *BRCA1* methylated tumors showed a statistically significant association with triple-negative status and exhibited poor outcome in terms of survival.

Genetic differences among different ethnicities/races may account for disparities in breast cancer susceptibility [3], [39]. Aberrant gene promoter methylation has also been shown to be affected by ethnicity in breast cancer [40]. Increased understanding of the genetic/epigenetic abnormality together with the ethnic factors/differences involved in the pathogenesis of breast cancer is crucial and may provide a basis for detection and treatment. Whether the phenomenon we observed in this study are due to ethnicity or etiology remained to be determine in larger studies that include breast cancer patients of different ethnicities/races.
