# Correction: Methylation of *BRCA1* promoter region is associated with unfavorable prognosis in women with early-stage breast cancer

**DOI:** 10.1371/journal.pone.0093778

**Published:** 2014-03-25

**Authors:** The PLOS ONE Staff

There is an error in the penultimate sentence of the Abstract. The correct sentence is: Multivariate analysis which incorporated variables of patients' age, tumor size, grade, and lymph node metastasis revealed that BRCA1 promoter methylation was associated with overall survival (p  =  0.027; hazard ratio, 16.38) and disease-free survival (p  =  0.003; hazard ratio, 12.19).

## References

[1] Hsu NC, Huang Y-F, Yokoyama KK, Chu P-Y, Chen F-M, et al (2013) Methylation of *BRCA1* Promoter Region Is Associated with Unfavorable Prognosis in Women with Early-Stage Breast Cancer. PLoS ONE 8(2): e56256 doi:10.1371/journal.pone.0056256 23405268 10.1371/journal.pone.0056256 PMC3566056

